# Characterizing cardiac involvement in amyloidosis using cardiovascular magnetic resonance diffusion tensor imaging

**DOI:** 10.1186/s12968-019-0563-2

**Published:** 2019-09-05

**Authors:** Alexander Gotschy, Constantin von Deuster, Robbert J. H. van Gorkum, Mareike Gastl, Ella Vintschger, Rahel Schwotzer, Andreas J. Flammer, Robert Manka, Christian T. Stoeck, Sebastian Kozerke

**Affiliations:** 1 Institute for Biomedical Engineering, University and ETH Zurich, Gloriastrasse 35, Zurich, 8092 Switzerland; 2 Department of Cardiology, University Hospital Zurich, Zurich, Switzerland; 3 Division of Medical Oncology and Hematology, University Hospital Zurich, Zurich, Switzerland; 4 Institute of Diagnostic and Interventional Radiology, University Hospital Zurich, Zurich, Switzerland

**Keywords:** Cardiovascular magnetic resonance imaging, Diffusion tensor imaging, Cardiac amyloidosis, Myocardial microstructure, Tissue characterization

## Abstract

**Background:**

In-vivo cardiovascular magnetic resonance (CMR) diffusion tensor imaging (DTI) allows imaging of alterations of cardiac fiber architecture in diseased hearts. Cardiac amyloidosis (CA) causes myocardial infiltration of misfolded proteins with unknown consequences for myocardial microstructure. This study applied CMR DTI in CA to assess microstructural alterations and their consequences for myocardial function compared to healthy controls.

**Methods:**

Ten patients with CA (8 AL, 2 ATTR) and ten healthy controls were studied using a diffusion-weighed second-order motion-compensated spin-echo sequence at 1.5 T. Additionally, left ventricular morphology, ejection fraction, strain and native T1 values were obtained in all subjects. In CA patients, T1 mapping was repeated after the administration of gadolinium for extracellular volume fraction (ECV) calculation. CMR DTI analysis was performed to yield the scalar diffusion metrics mean diffusivity (MD) and fractional anisotropy (FA) as well as the characteristics of myofiber orientation including helix, transverse and E2A sheet angle (HA, TA, E2A).

**Results:**

MD and FA were found to be significantly different between CA patients and healthy controls (MD 1.77 ± 0.17 10^− 3^ vs 1.41 ± 0.07 10^− 3^ mm^2^/s, *p* <  0.001; FA 0.25 ± 0.04 vs 0.35 ± 0.03, *p* <  0.001). MD demonstrated an excellent correlation with native T1 (*r* = 0.908, *p* <  0.001) while FA showed a significant correlation with ECV in the CA population (*r* = − 0.851, *p* <  0.002). HA exhibited a more circumferential orientation of myofibers in CA patients, in conjunction with a higher TA standard deviation and a higher absolute E2A sheet angle. The transmural HA slope was found to be strongly correlated with the global longitudinal strain (*r* = 0.921, *p* < 0.001).

**Conclusion:**

CMR DTI reveals significant alterations of scalar diffusion metrics in CA patients versus healthy controls. Elevated MD and lower FA values indicate myocardial disarray with higher diffusion in CA that correlates well with native T1 and ECV measures. In CA patients, CMR DTI showed pronounced circumferential orientation of the myofibers, which may provide the rationale for the reduction of global longitudinal strain that occurs in amyloidosis patients. Accordingly, CMR DTI captures specific features of amyloid infiltration, which provides a deeper understanding of the microstructural consequences of CA.

**Electronic supplementary material:**

The online version of this article (10.1186/s12968-019-0563-2) contains supplementary material, which is available to authorized users.

## Introduction

Amyloidosis is a multi-system disease that is caused by the synthesis and accumulation of unstable and misfolded proteins leading to a loss of normal tissue architecture and function [1]. While almost every organ can be affected by amyloid deposition, the prognosis of patients is mainly determined by the occurrence and extent of myocardial involvement [2–4]. There are various types of amyloid that differ in the source and nature of the misfolded precursor protein. Of those, light chain associated amyloidosis (AL) and transthyretin amyloid amyloidosis (ATTR) are the two main types of amyloidosis that affect the heart. The infiltration of amyloid to the heart leads to myocardial thickening and diastolic dysfunction resulting in heart failure.

For the assessment of cardiac amyloidosis (CA), cardiac imaging plays a fundamental role [5]. Besides myocardial thickening, echocardiography often shows a reduction of global longitudinal strain, which typically spares the apical segments [6]. Non-invasive characterization of the myocardial tissue of amyloidosis patients is the strength of cardiovascular magnetic resonance (CMR). In particular, the extent and distribution of late gadolinium enhancement (LGE) has been shown to provide independent information on outcome even after adjustment for known prognostic factors [7]. CA is also an ideal application for newer mapping techniques as it leads to excessively high T1- and extracellular volume (ECV) [8, 9]. Notably, in AL CA patients, ECV is a proven predictor of mortality with an up to four-fold increased likelihood of death for ECV > 0.45 over a median follow-up period of 23 months [10]. Both LGE and ECV, however, rely on the application of gadolinium-based contrast agents (GBCA), which is often limited in amyloidosis patients due to severely reduced renal function.

Recently, CMR diffusion tensor imaging (DTI) emerged as a promising method for the determination of myocardial fiber orientation [11–13] and tissue characterization without the need for GBCA. It allows to investigate water diffusion within the tissue and to derive additional scalar metrics, such as mean diffusivity (MD) and fractional anisotropy (FA) for the quantification of structural integrity [14, 15]. Alterations of cardiac microstructure have been described in patients with dilated cardiomyopathy (DCM), myocardial infarction and hypertrophic cardiomyopathy (HCM) [16–18]. In CA, however, the effect of the double harm resulting from the combination of amyloid infiltration with direct cardio-toxicity of abnormal proteins [19] on the myocardial structure and its potential impact on diffusion properties is yet unknown.

The objective of this study was to determine the consequences of amyloid infiltration on myocardial microstructure in-vivo using CMR DTI and to investigate their effect on myocardial function. Scalar diffusion and relaxation metrics (MD/FA and native T1/ECV, respectively) were correlated in CA patients and controls. In addition, directional diffusion information was assessed to reveal changes in myofiber orientations and compared to parameters of left ventricular (LV) function.

## Methods

### Study design

Between March 2017 and June 2018, 12 patients with AL or ATTR CA (63 ± 11 years, 3 female), referred to the local outpatient clinic, and 10 healthy, age-matched controls (62 ± 11 years, 4 female) were prospectively enrolled. Exclusion criteria were cardiovascular disease other than CA, kidney failure with estimated glomerular filtration rate (eGFR) < 30 ml/min/1.76 m^2^ and the standard exclusion criteria for CMR [20]. Height and weight of all study subjects were recorded to calculate the body surface area (BSA). All patients had histologically proven (positive Congo red staining of endomyocardium, abdominal fat, kidney, rectum or bone marrow biopsies) amyloidosis and echocardiographic or CMR findings typical for cardiac involvement. Imaging was performed on a clinical 1.5 T system (Achieva, Philips Healthcare, Best, The Netherlands) equipped with a 5-channel cardiac receiver array. Prior to imaging, written informed consent was obtained from all subjects. The study protocol was approved by the ethics committee of the canton of Zurich; it allowed the administration of GBCA only in the CA patient group.

### CMR data acquisition

Cardiac function was assessed by a contiguous stack of balanced steady-state free precession (bSSFP) short-axis cine images covering the entire LV and bSSFP long-axis cine images in 2-chamber, 3-chamber and 4-chamber orientations. According to the cine images, systolic quiescent time points were determined on a per subject basis.

Diffusion-weighted imaging was performed with a single-shot spin-echo sequence utilizing second-order motion compensated diffusion sensitizing gradients in combination with an echo planar imaging (EPI) readout [21, 22]. The sequence was electrocardiogram (ECG)-triggered to mid-systole systole (65% of peak systole) and three imaging planes were placed in short-axis view orientation at apical, mid-ventricular and basal level LV using a reduced field-of-view (FOV) technique [23]. Diffusion weighting was encoded along nine and three directions with a b-value of 450 and 100 s/mm^2^, respectively [24]. Data acquisition was performed during free breathing with respiratory navigator-based slice tracking [25]. The imaging parameters are listed in Table 1.

**Table 1:** CMR Scan Parameters

	DTI	T1 Mapping	LGE
Spatial Resolution [mm^3^]	2.5 × 2.5 × 8	1.2 × 1.2 × 8	1.6 × 1.6 × 10
FOV [mm^2^]	230 × 105	300 × 300	360 × 460
TR	3 beats	2.5 ms	3.5 ms
TE [ms]	76	1	1.7
Flip Angle [°]	90	35	15
Number of Averages	12	1	1
Respiratory Mode	Free Breathing	Breath Hold	Breath Hold
Approx. Scan Time [min.]	8–10 min	1–2 min	1 min

*DTI* diffusion tensor imaging, *FOV* field of view, *LGE* late gadolinium enhancement, *TE* echo time, *TR* repetition time

T1 mapping was performed using a modified Look-Locker inversion (MOLLI) sequence with a total of eight T1 weighted images split into a set of five and three images acquired after inversion, separated by three heartbeats of recovery (5-(3)-3 scheme) [26]. The imaging planes were at the same slice location as the DTI scan. In all subjects, native T1 maps were acquired. For ECV calculation, T1 mapping was repeated in the patient group 20 min after a bolus injection of 0.2 mmol/kg gadolinium-based contrast agent (Gadovist, Bayer Schering, Berlin, Germany). The hematocrit was measured in all patients within 2 h of CMR. LGE images were acquired with an inversion-recovery sequence approximately 10 min after the bolus injection.

### CMR data analysis

Standard measurements of LV size and function including LV ejection fraction (LVEF), left ventricular mass index (LVMi) and intraventricular septal wall thickness (IVS) were obtained using the GTVolume software package (GyroTools LLC, Zurich, Switzerland). The LV end-systolic global longitudinal strain (GLS) and global circumferential strain (GCS) were calculated using a certified CMR feature tracking evaluation software (2 D CPA MR, Cardiac Performance Analysis MR Version 4, TomTec Imaging Systems, Unterschleissheim, Germany) on the long- and short-axis bSSFP images.

Prior to diffusion tensor calculation and T1 fitting, the individual MOLLI and DTI images were registered using a non-rigid groupwise image registration method [27] for compensation of residual motion. DTI analysis was performed on MD, FA, helix angle (HA), transverse angle (TA) and absolute E2A sheet angle [16, 28]. A detailed description of the DTI parameters is provided as Additional file 1. For calculating the HA slope, a linear regression of the transmural course of the helix angles between the endocardium and epicardium was performed, excluding boundary pixels at the endo- and epicardium. Angular diffusion metrics were evaluated in four radial and circumferential sectors of the left ventricle and averaged across all three slices. Mean LV signal-to-noise ratio (SNR) of the b = 100 s/mm^2^ image was determined in both patient groups for quality control. The scalar diffusion metrics MD and FA as well as native T1 and ECV, were evaluated in a single septal region-of-interest (ROI) on the midventricular short-axis slices in accordance with current clinical recommendations [29]. ECV was computed based on the collected haematocrit, native and enhanced T1 values in the myocardium and the blood pool.

### Statistical analysis

All data are expressed as mean ± SD. Statistical analysis was conducted using MedCalc software (MedCalc 17.9.7, MedCalc Software bvba, Ostend, Belgium). Differences in diffusion and relaxation metrics between patients and controls were assessed by unpaired two-tailed t-tests. To determine statistical associations between parameters, Pearson correlation analyses were performed. In addition, the diagnostic accuracy of native T1, MD and FA for detecting cardiac amyloidosis compared to healthy controls was examined by ROC analysis. A *p*-value less than 0.05 was considered statistically significant.

## Results

### Patient characteristics

Data from two of 12 CA subjects were excluded, one for difficulty with breath-holding and one for technical reasons. Ten CA patients (60 ± 11 years, 7 male) were hence included in the analysis. Eight patients suffered from AL-amyloidosis while two had ATTR-amyloidosis (one wild type, one heterozygous mutation c.238A > G). The diagnosis of CA was confirmed by endomyocardial biopsy (*n* = 4) or the combination of typical imaging findings (echocardiography or CMR) with proven amyloid deposition in bone marrow (*n* = 3), kidney (*n* = 1), rectum (*n* = 1) or abdominal fat (*n* = 1) biopsies. The renal function was normal in two patients, while there were five patients with mildly and three patients with moderately reduced renal function (eGFR: 70 ± 21 ml/min). The age- and sex-matched control group also showed similar BSA characteristics. The IVS (14.4 ± 2.6 vs 8.0 ± 1.3 mm, *p* < 0.001) and the LVMi (79 ± 19 vs 44 ± 7 g/m^2^, *p* < 0.001) were significantly elevated in CA patients. While GLS was significantly impaired in CA patients (− 16.6 ± 3.3 vs − 22.4 ± 1.9%, *p* < 0.001), GCS (− 26.9 ± 5.5 vs − 29.1 ± 3.1%, *p* = 0.30) and LVEF (64 ± 6 vs 61 ± 4%, *p* = 0.20) exhibited no significant difference between the groups. Table 2 contains the baseline characteristics of both groups.

**Table 2:** Baseline characteristics of the Study Population

	CA Patients *N* = 10	Healthy Controls *N* = 10	*p*-value
Age [years]	60 ± 11	62 ± 11	0.79
Male (%)	7 (70)	6 (60)	0.73
BSA [m^2^]	1.80 ± 0.13	1.85 ± 0.18	0.50
AL-Amyloidosis	8 (80)		
ATTR-Amyloidosis	2 (20)		
IVS [mm]	14.4 ± 2.6	8.0 ± 1.3	< 0.001
LVEDV [ml]	134 ± 25	133 ± 20	0.97
LVMi [g/m2]	79 ± 19	44 ± 7	< 0.001
LVEF [%]	64.0 ± 6.3	60.7 ± 3.9	0.20
GLS [%]	−16.6 ± 3.3	−22.4 ± 1.9	< 0.001
GCS [%]	−26.9 ± 5.5	−29.1 ± 3.1	0.30

Data are mean ± SD or number of patients (%)

*AL* amyloid light chain, *ATTR* transthyretin-associated amyloidosis, *BSA* body surface area, *CA* cardiac amyloidosis, *GCS* global circumferential strain, *GLS* global longitudinal strain, *IVS* interventricular septum thickness in diastole, *LVEDV* left ventricular end-diastolic volume, *LVEF* left ventricular ejection fraction, *LVMi* left ventricular mass indexed to body surface area

### Comparison of scalar DTI parameters with ECV and native T1

**Fig. 1:**
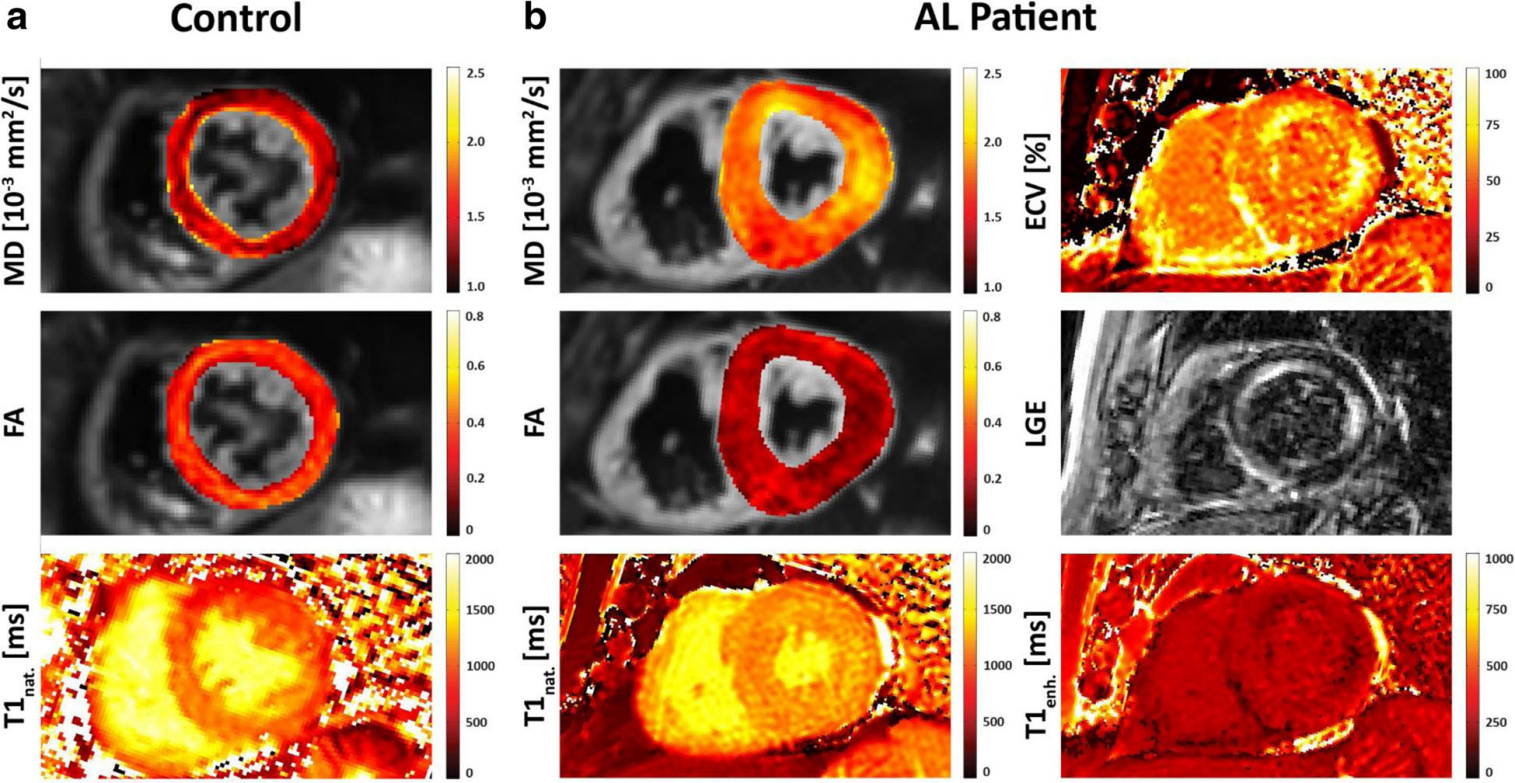
Representative mean diffusivity (MD), fractional anisotropy (FA) and native T1 maps for a healthy control (**a**) and a light chain (AL) amyloidosis patient (**b**). Enhanced T1 mapping, extracellular volume fraction (ECV) mapping and late gadolinium enhancement (LGE) CMR imaging was only performed in the cardiac amyloid (CA) patient. It can be appreciated, that the areas of signal hyperenhancement in LGE and elevated ECV correspond well with regions of high MD and low FA in the parameter maps derived from CMR diffusion tensor imaging (DTI) in the CA patient

Midventricular septal reference values, obtained in the healthy controls were 1.41 ± 0.07 10^− 3^ mm^2^/s for MD, 0.35 ± 0.03 for FA and 1020 ± 42 ms for native T1. In the CA group, a significantly higher native T1 (1136 ± 61 ms, *p* < 0.001), highly elevated MD (1.77 ± 0.17 10^-3^ mm^2^/s, *p* < 0.001) and significantly reduced FA (0.25 ± 0.04, *p* < 0.001) were found when comparing with the healthy control group. ECV in the CA group was elevated to 0.50 ± 0.14. Figure 1 depicts representative mid-ventricular slices of MD, FA and native T1 of a healthy control and an AL-amyloidosis case with additional ECV and LGE images in the CA patient. Comparing the different modalities within the CA patient, it can be appreciated that the areas of LGE hyperenhancement and elevated ECV correspond well with the regions of high MD and low FA in the parameter maps derived from DTI.

**Fig. 2:**
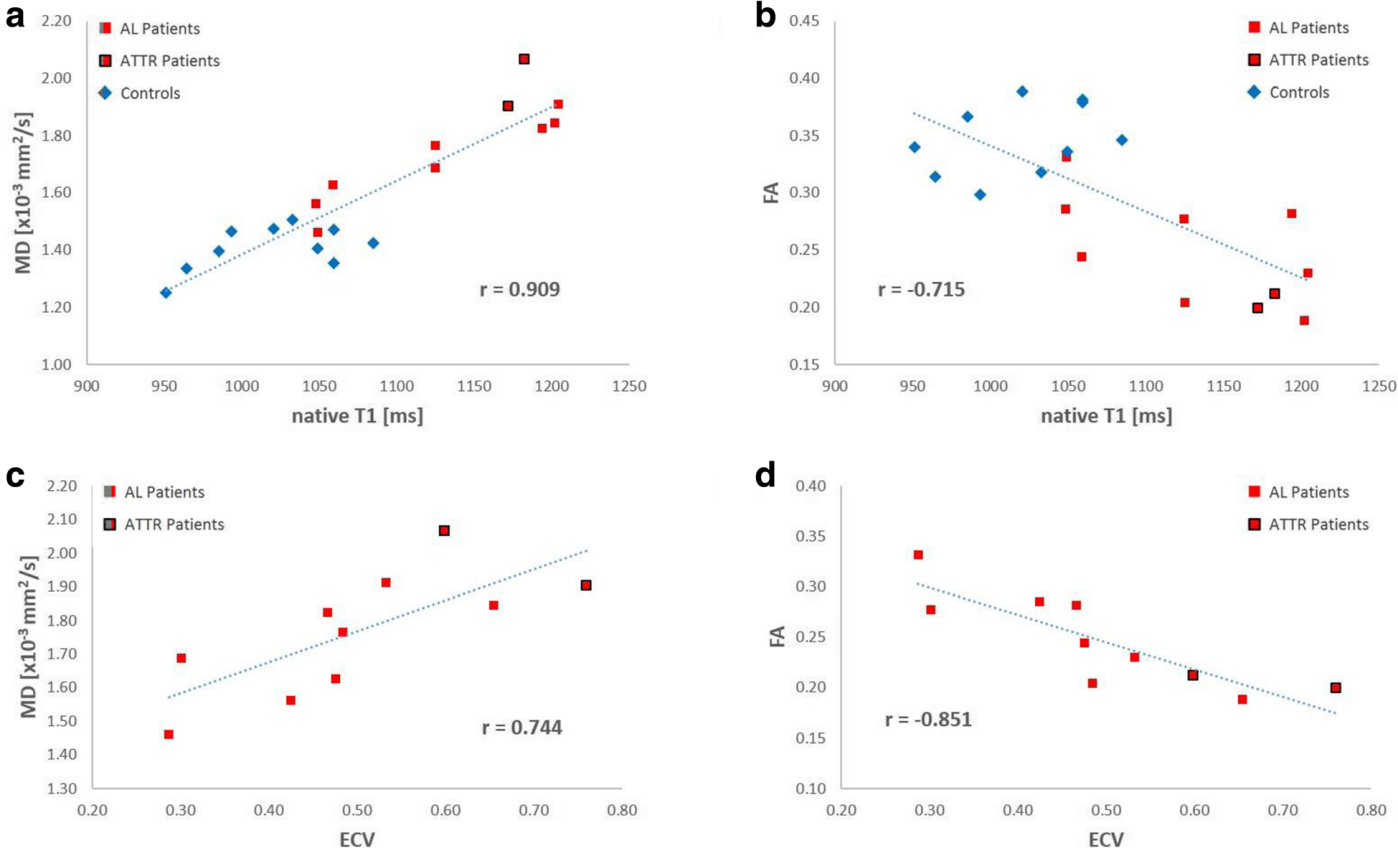
Correlation plots for MD and FA with native T1 and ECV. Native T1 shows an excellent correlation with MD (**a**) and a moderate but still significant negative correlation with FA (**b**). Within the patient population, MD is also correlated with ECV (**c**) but FA exhibits a higher correlation with ECV (**d**)

Direct comparison between native T1 values versus MD over the whole study population demonstrates an excellent correlation between the two parameters (*r* = 0.909, 95% CI [0.778, 0.963], *p* < 0.001, Fig. 2a). A weaker, but still significant negative correlation was also found between native T1 and FA (*r* = − 0.715, 95% CI [− 0.879, − 0.398]; *p* < 0.001; Fig. 2b). The analysis of correlation between DTI-based parameters and ECV was limited to the CA group. It exhibited a fair correlation between ECV and MD (*r* = 0.744, 95% CI [0.216, 0.936]; *p* = 0.014; Fig. 2c) and a strong negative correlation between ECV and FA (*r* = − 0.851, 95% CI [− 0.964, − 0.477]; *p* < 0.002; Fig. 2d).

For the differentiation between healthy controls and CA patients, the scalar DTI parameters exhibit promising accuracy with an area under the curve (AUC) of 0.96 [95% CI, 0.77–1.00] for MD and 0.97 [95% CI, 0.78–1.00] for FA which were not significantly different from the diagnostic performance of native T1 (AUC 0.91 [95% CI, 0.69–0.99]). Additional file 2: Figure S2 provides detailed information on the AUC analysis (Additional file 2).

### Evaluation of myocardial microstructure in cardiac amyloidosis

**Fig. 3:**
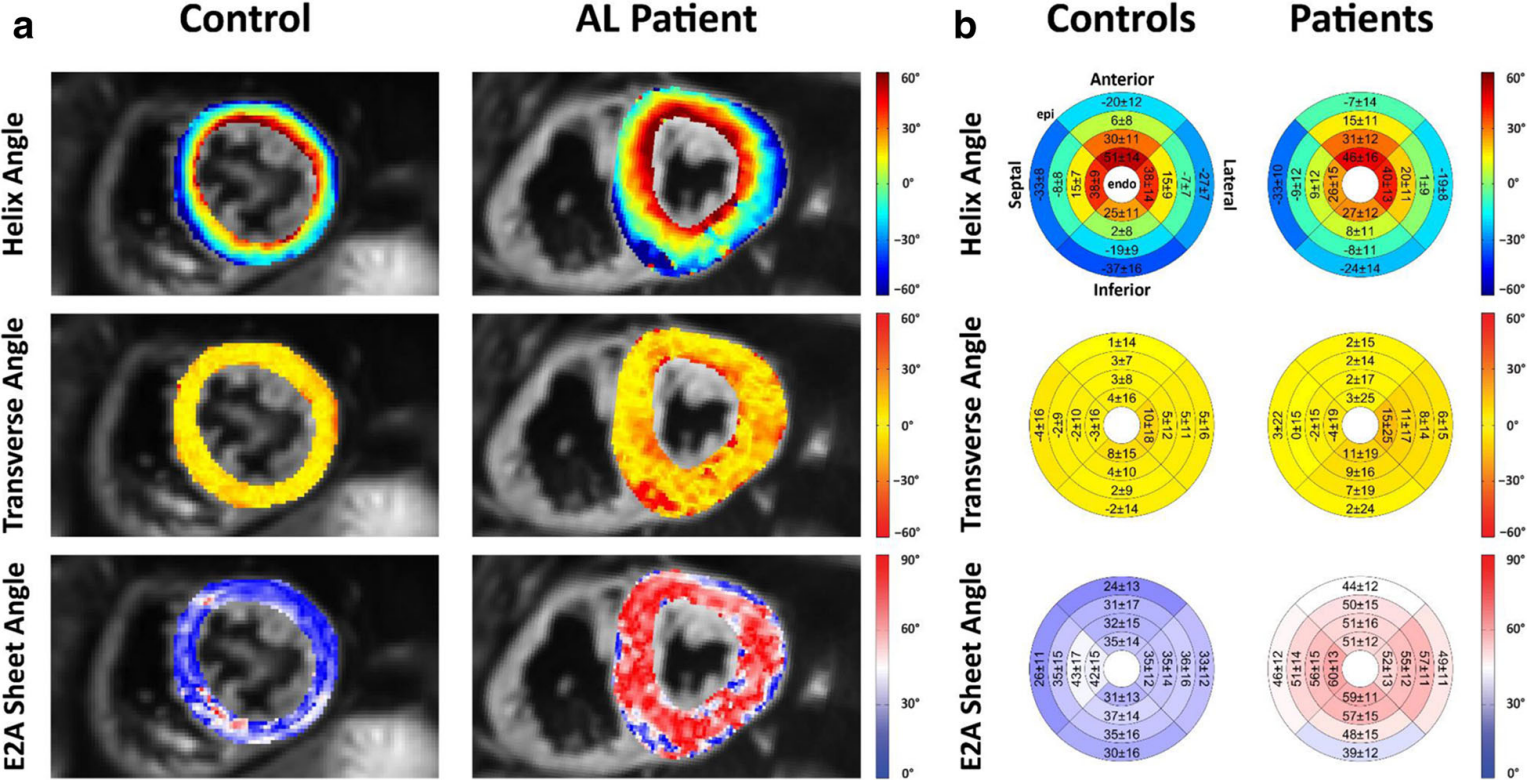
Example helix angle (HA), transverse angle (TA) and sheet angle (E2A) map for a healthy control subject and an AL-amyloidosis patient (**a**). Image (**b**) shows Bullseye plots for HA, TA and E2A across all subjects for each cohort. Reported values are mean ± SD

Figure 3a depicts the comparison of the HA, TA and absolute E2A sheet angle of a representative CA patient and a healthy control. At first sight, despite the thicker myocardium in CA patients, the distribution of HA seems similar between both groups (Fig. 3a first row). Quantitative evaluation, however, reveals that the transmural HA slope is significantly reduced in CA patients (− 0.83 ± 0.16 °/% transmural depth (tmd) vs − 1.02 ± 0.14 °/%_tmd_; *p* < 0.001). In case of the patients, this leads to a reduced longitudinal orientation of fibers in the epicardial and endocardial layers compared to the controls as shown in the bullseye plots in Fig. 3b first row.

The TA is close to zero degrees in both groups with no significant difference between CA patients and healthy controls (4.5 ± 4.6 vs 2.5 ± 3.6°, *p* = 0.07). The standard deviation per segment, however, is significantly larger in the CA patients (18.2 ± 6.6 vs 12.7 ± 2.6°, *p* < 0.001) indicating a higher level of deviation from the circumferential alignment of the myocardial fibers found in healthy subject myocardium. Representative TA maps are shown in Fig. 3a middle row. The higher TA standard deviation can be appreciated by the more irregular appearance of the TA map in the CA patient and the quantitative overview in Fig. 3b middle row.

The most prominent difference in the myocardial microstructure between CA patients and healthy controls becomes evident in the comparison of the absolute E2A sheet angle. Patients exhibited higher mean absolute E2A sheet angles in every segment with values approximately 20° greater than those of healthy subjects (52.9 ± 7.5 vs 33.9 ± 9.5°, *p* < 0.001, Fig. 3ab lower row). Average SNR of a single b = 100 s/mm^2^ image was comparable between patients and controls (12.0 ± 3.0 vs 12.5 ± 3.6, *p* = 0.62).

### Correlation between myocardial microstructure and left ventricular function

**Fig. 4:**
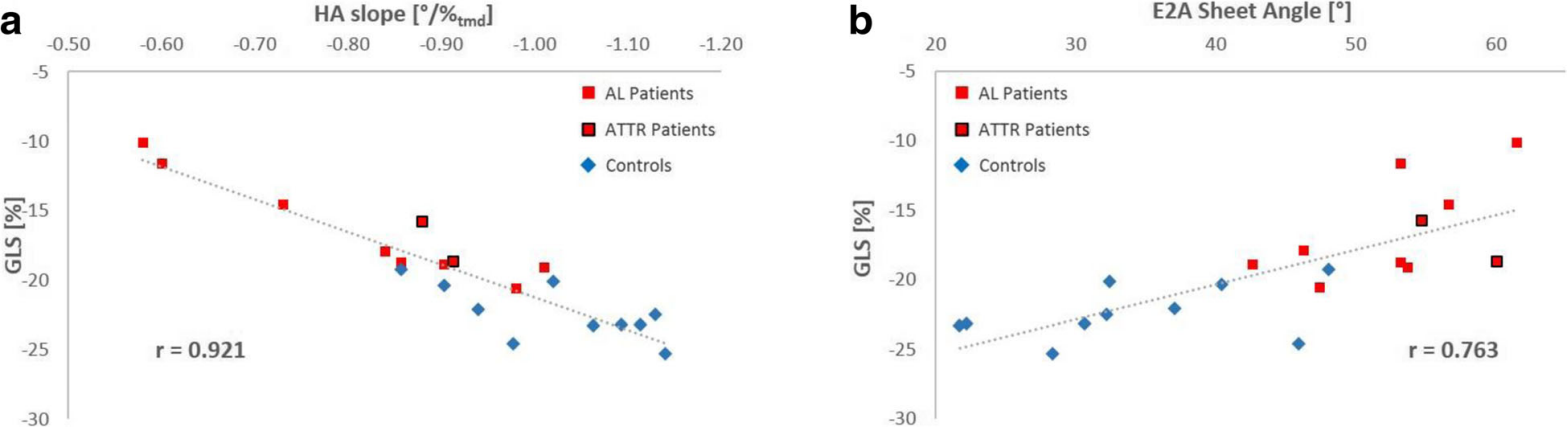
Correlation plots for GLS with HA slope and E2A sheet angle. The HA slope shows an excellent correlation with GLS (**a**) while a moderate but still significant correlation between GLS and E2A could be observed (**b**)

HA slope and E2A sheet angle were correlated against LVEF, GLS and GCS. A strong correlation was found between HA slope and GLS (*r* = 0.921, 95% CI [0.809, 0.969]; *p* < 0.0001; Fig. 4a), indicating impaired longitudinal function with a flatter transmural HA gradient. A weaker, but still significant correlation could be observed between E2A sheet angle and GLS (*r* = 0.763, 95% CI [0.483, 0.901]; *p* = 0.0001; Fig. 4b). LVEF and GCS exhibited no significant correlation with any parameter describing the myocardial microstructure.

## Discussion

This study assessed the value of cardiac DTI for the evaluation of patients with CA and investigated the effects of amyloid deposition on myocardial microstructure.

The scalar DTI metrics of MD and FA were found to be significantly altered by CA. In comparison with native T1, which was also elevated in CA patients in agreement with previous studies [10, 30], MD and FA exhibited a comparable AUC to identify CA patients indicating potential for similar diagnostic accuracy. The correlation between MD and native T1 suggests that the pathomechanism of amyloid infiltration affects both parameters to a similar extent. While native T1 is a useful diagnostic marker for CA, recent studies have shown that it is inferior to ECV as an independent predictor of prognosis in AL-amyloidosis as well as ATTR-amyloidosis patients [30, 31]. However, the significant number of amyloidosis patients suffering from renal failure stresses the need for a gadolinium-free substitute for ECV with potential prognostic value. The robust correlation between FA and ECV found in the CA patients justifies expectations, that FA may have potential to serve as such a substitute for ECV. This warrants future trials to investigate the diagnostic accuracy of FA and MD for amyloidosis in particular against other cardiomyopathies with a HCM phenotype. The existence of a tight functional relation between FA and ECV is also suggestive from a microstructural perspective, since the scalar CMR DTI parameters describe the range and anisotropy of myocardial water movement, which is mainly determined by the tissue-specific presence of spatially ordered cellular compartments within the myocardium [32]. Our findings are supported by previous studies, which have demonstrated that quantitative measures of diffusivity derived from CMR DTI capture similar properties as LGE and ECV in myocardial infarction and HCM [33, 34].

Time efficiency is an essential criterion for clinical practice under the economic constraints of healthcare systems. From this point of view, measurement durations of approximately ten minutes seem unreasonably long and insufficient for daily routine in CMR imaging. However, the presented sequence acquired three slices, two of which, namely the basal and apical slice were only used for the determination of myocardial microarchitecture. The MD and FA values were only assessed in the mid-ventricular slice as recommended in the current consensus document [29]. Limiting the acquisition to a single mid-ventricular slice would reduce the measurement time by approximately 25% assuming a 1.5 T CMR system and a heart rate of 60 beats per minute. In addition, replacing LGE and ECV with DTI would also eliminate the 10–15 min waiting period before LGE and ECV [35, 36] imaging and could ultimately lead to a saving of time.

Our investigation of myocardial architecture provided new insights into the microstructural alterations due to interstitial amyloid deposition. The most prominent observation of significantly increased absolute E2A sheet angle is the structural equivalent of a more systolic conformation of the myocardial sheetlets. The correlation between E2A sheet angle and GLS may indicate that, at a microstructural level, a pronounced systolic sheetlet configuration might be associated with a limited ability to rotate to a relaxed conformation in diastole. Similar observations have been made in HCM patients [17], indicating that despite fundamentally different pathogenesis, comparable structural alterations may cause the primary diastolic heart failure observed in both diseases. The difference in HA between health and disease, however, has not yet been described. The significantly lower HA slope of CA patients results in a reduced transmural HA gradient with a less longitudinal orientation of the myocardial fibers. The strong correlation which we observed between the HA slope and the GLS indicates, that the more circumferential myofiber orientation may be the microstructural determinant for the loss of longitudinal function in amyloidosis patients. This finding seems not to be a common feature of all hypertrophic phenotype cardiomyopathies as Nielles-Vallespin et al. described similar HA configuration in HCM patients and healthy controls [17]. In agreement with the hypothesis of the HA slope being a determinant for longitudinal function, a recent study by Pagourelias et al. demonstrated that the GLS of CA patients is significantly lower compared to HCM patients with identical myocardial wall thickness [37]. In addition to the microstructural alterations, the direct cardio-toxicity of amyloid deposition may also contribute to a loss of myocardial function in CA patients [19].

It remains unclear whether the relation between HA slope and GLS is linear over a wider range. A previous study by von Deuster et al. [16] in DCM patients reported similar HA slope in controls and patients with mildly reduced LVEF, but two patients with advanced heart failure showed reduced GLS and increased HA slope. In synopsis, these findings support the hypothesis of a U-shaped relation between HA slope and GLS with an ideal HA slope around 1°/%_tmd_ and impaired longitudinal function caused by deviations to significantly higher and lower HA slope values. The different constellations of DTI parameters observed in HCM, DCM and amyloidosis raise the hope that future research may identify disease specific patterns of DTI parameters, which would be an advantage over rather unspecific parameters such as native T1 that is elevated in a multitude of cardiac diseases. The presence of subtype specific patterns of the DTI parameters for AL- and ATTR-amyloidosis should be investigated, in particular in comparison to nuclear imaging techniques where radiotracers used for technetium-labeled bone scintigraphy are found to have high sensitivity for ATTR [38], while ^18^F-florbetaben-PET has recently been shown to have a high sensitivity for AL-amyloidosis [39].

In contrast to HA and E2A, the transverse angle does not seem to provide relevant insights into disease specific pathologies as it is distributed around 0° in health and disease. However, the elevated standard deviation of TA that we observed in amyloidosis patients has previously already been observed in DCM patients [16] and may therefore be a surrogate for myocyte disarray of any cause. A bias of the TA distribution due to low SNR, which has been observed in previous studies [24], could be excluded in our work since SNR values were not different between the amyloidosis and control cohorts.

### Limitations

A limitation of the present work is the small sample size of CA patients with different types of the disease. In particular the patient number is insufficient to investigate potential effects of the different pathophysiologies of AL- and ATTR-amyloidosis on the myocardial microstructure. In addition, the lack of GBCA application in healthy controls, due to constraints from ethical approval constitutes a limitation. However, despite those limitations, our patient population already showed promising diagnostic properties of CMR DTI based parameters warranting future research in a larger population of CA patients. In contrast to previous studies using STEAM based DTI methods [40], this study was limited to investigate the mid-systolic configuration of the myocardium and did not assess a diastolic state.

## Conclusion

The scalar CMR DTI parameters MD and FA in CA patients are significantly different from healthy controls, allowing to assess the amyloid induced microstructural disarray without the use of GBCA. The major differences in myofiber orientation are a significantly increased E2A sheet angle and a reduced HA slope in CA patients. Finally, the strong correlation between HA slope and GLS indicates, that the more circumferential orientation of the myofibers may provide the rationale for the reduction of CLS that occurs in CA.

## Additional files

Additional file 1: **Figure S1.** (PDF 482 kb)

Additional file 2: **Figure S2.** (PDF 61 kb)

## Data Availability

The datasets used and analyzed during the current study are available from the corresponding author on reasonable request.

## Abbreviations

AL: Amyloid light chain
ATTR: Transthyretin-associated amyloidosis
AUC: Area under the curve
BSA: Body surface area
bSSFP: balanced steady-state free precession
CA: Cardiac amyloidosis
CMR: Cardiovascular magnetic resonance
DCM: Dilated cardiomyopathy
DTI: Diffusion tensor imaging
E2A: E2A sheet angle
ECG: Electrocardiogram
ECV: Extracellular volume
EDV: End-diastolic volume
EGFR: Estimated glomerular filtration rate
EPI: Echo planar imaging
FA: Fractional anisotropy
FOV: Field of view
GBCA: Gadolinium based contrast agent
GCS: Global circumferential strain
GLS: Global longitudinal strain
HA: Helix angle
HCM: Hypertrophic cardiomyopathy
IVS: Interventricular septum thickness
LGE: Late gadolinium enhancement
LV: Left ventricle/left ventricular
LVEF: Left ventricular ejection fraction
LVMi: Left ventricular indexed mass
MD: Mean diffusivity
MOLLI: Modified Look Locker inversion recovery
ROC: Receiver operating characteristic
RV: Right ventricle/right ventricular
RVEF: Right ventricular ejection fraction
SD: Standard deviation
SV: Stroke volume
TA: Transverse angle
TE: Echo time
TR: Repetition time
