# Supplementary material for: Characterizing cardiac involvement in amyloidosis using cardiovascular magnetic resonance diffusion tensor imaging
Source: J Cardiovasc Magn Reson. 2019 Sep 5;21:56. doi: 10.1186/s12968-019-0563-2 (PMC6727537; doi:10.1186/s12968-019-0563-2)
Supplement: Supplementary file 1 — Figure S1. (PDF 482 kb) [file 12968_2019_563_MOESM1_ESM.pdf]

Supplemental Figure 1

a)

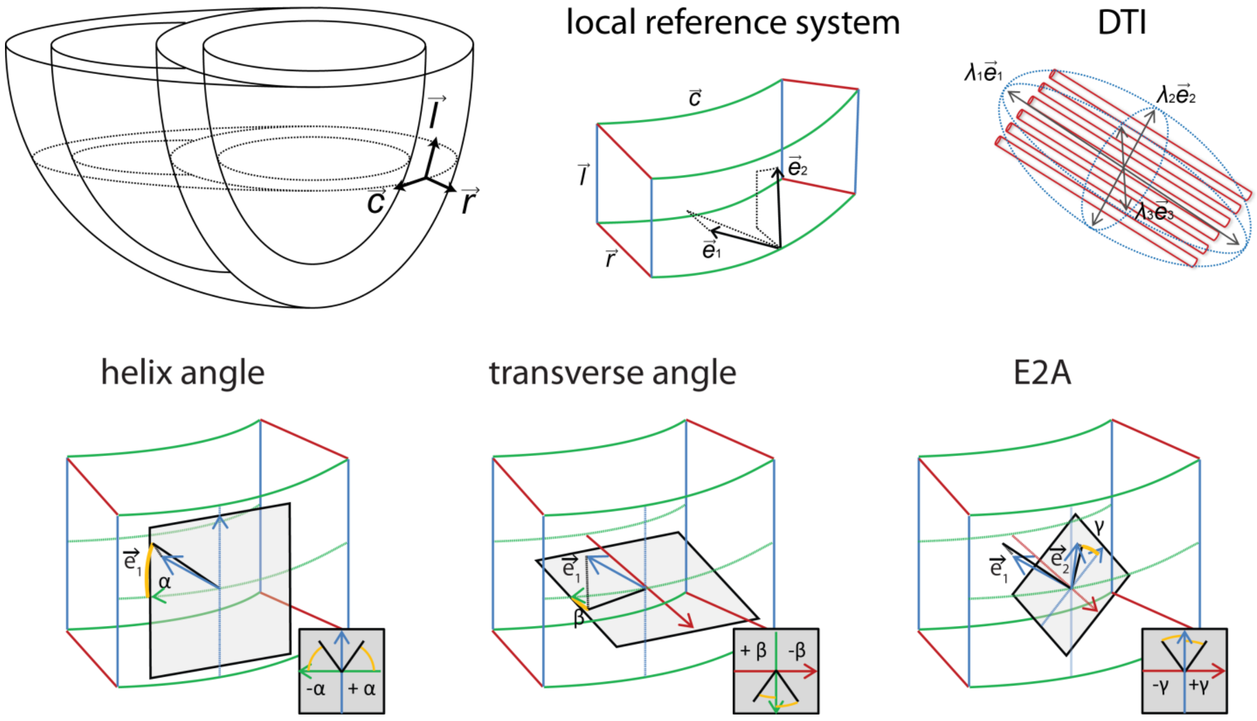

b)

$$MD = \frac{\lambda_1+\lambda_2+\lambda_3}{3} \qquad\qquad FA = \sqrt{\frac{1}{2}} * \frac{\sqrt{(\lambda_1-\lambda_2)^2+(\lambda_1-\lambda_3)^2+(\lambda_2-\lambda_3)^2}}{\sqrt{\lambda_1^2+\lambda_2^2+\lambda_3^2}}$$

Supplemental Figure 1a) describes the definition of helix, transverse and E2A angulation with respect to a local reference system. A detailed description is found in (1). For each pixel, a local reference system is computed following the curvature of the left ventricle. It is spanned by three orthogonal vectors in radial $\vec{r}$, circumferential $\vec{c}$ and longitudinal direction $\vec{l}$. Furthermore, for each pixel within the myocardium, the diffusion tensor is computed from the DTI measurements and its eigenbasis $\overrightarrow{e_1} to\ \overrightarrow{e_3}$. From histological studies, it has been shown, that the first eigenvector $\overrightarrow{e_1}$ follows the principal fibre orientation (2), the second $\overrightarrow{e_2}$ is aligned within the sheetlet (orthogonal to $\overrightarrow{e_1}$) (3) and the third $\overrightarrow{e_3}$ being orthogonal to $\overrightarrow{e_1}$ and $\overrightarrow{e_2}$ by definition represents the sheetlet normal. Having identified the principal directions of the diffusion tensor as well as the local reference system microstructural angulation is defined as follows:

Helix angle: After projection of the first eigenvector $\overrightarrow{e_1}$ onto the longitudinal-circumferential plane spanned by $\vec{l}$ and $\vec{c}$, the helix angle α is computed between the circumferential direction $\vec{c}$ and the projected vector.

Transverse angle: After projection of the first eigenvector $\overrightarrow{e_1}$ onto the radial-circumferential plane spanned by $\vec{r}$ and $\vec{c}$, the transverse angle β is computed between the circumferential direction $\vec{c}$ and the projected vector.

E2A sheet angle: First the myocyte cut plane is defined orthogonally to the projection of first eigenvector $\overrightarrow{e_1}$ onto the longitudinal-circumferential. The second eigenvector $\overrightarrow{e_2}$ is consequently projected onto this virtual cut plane. The E2A sheet angle γ is defined as angle between the projected vector and the intersecting line of the virtual cut plane with the longitudinal-circumferential plane spanned by $\vec{l}$ and $\vec{c}$.

Supplemental Figure 1b) shows the definitions of mean diffusivity (MD) and fractional anisotropy (FA) which are scalar parameters that are derived from the eigenvalues (λ_1, λ_2 and λ_3) corresponding to the three eigenvectors. Thereby, MD provides an average of all three eigenvalues and can be interpreted as measure of the average diffusion of water molecules in the myocardium, independent of direction. Its value depends on the imaging sequence used as well as the underlying structure. In tissue with a large number of boundaries close to each other such as intact cell membranes, the free path of water molecules is restricted leading to a reduction in measured MD. In our experiment a higher MD reflects a larger mean free pathway, with less water molecules having reached a boundary along their random walk, due to e.g. larger spaces between cell membranes. It has been shown in previous studies that ischemic or hypertrophic cardiomyopathies lead to increased diffusion in the myocardium.

Fractional anisotropy (FA) quantifies the relative difference between the eigenvalues and can be interpreted as a measure of the extent to which diffusion is confined towards a predominant direction. FA can reach values between 0 and 1, while 0 represents isotropic free diffusion in all directions and 1 represents fully anisotropic diffusion restricted to one predominant direction. Healthy myocardium is highly organized in fibres and sheetlets and, therefore, represents an anisotropic structure. Decreasing anisotropy can indicate a breakdown of the myocardial structure.

1. Stoeck CT, von Deuster C, Fleischmann T, Lipiski M, Cesarovic N, Kozerke S. Direct comparison of in vivo versus postmortem second-order motion-compensated cardiac diffusion tensor imaging. Magn. Reson. Med. 2017;00. doi: 10.1002/mrm.26871.

2. Holmes A, Scollan DF, Winslow RL. Direct histological validation of diffusion tensor MRI in formaldehyde-fixed myocardium. Magn. Reson. Med. 2000;44:157–161.

3. Kung GL, Nguyen TC, Itoh A, Skare S, Ingels NB, Miller DC, Ennis DB. The presence of two local myocardial sheet populations confirmed by diffusion tensor MRI and histological validation. J. Magn. Reson. Imaging 2011;34:1080–91.
