# Supplementary material for: Characterizing cardiac involvement in amyloidosis using cardiovascular magnetic resonance diffusion tensor imaging
Source: J Cardiovasc Magn Reson. 2019 Sep 5;21:56. doi: 10.1186/s12968-019-0563-2 (PMC6727537; doi:10.1186/s12968-019-0563-2)
Supplement: Supplementary file 2 — Figure S2. (PDF 61 kb) [file 12968_2019_563_MOESM2_ESM.pdf]

Supplemental Figure 2

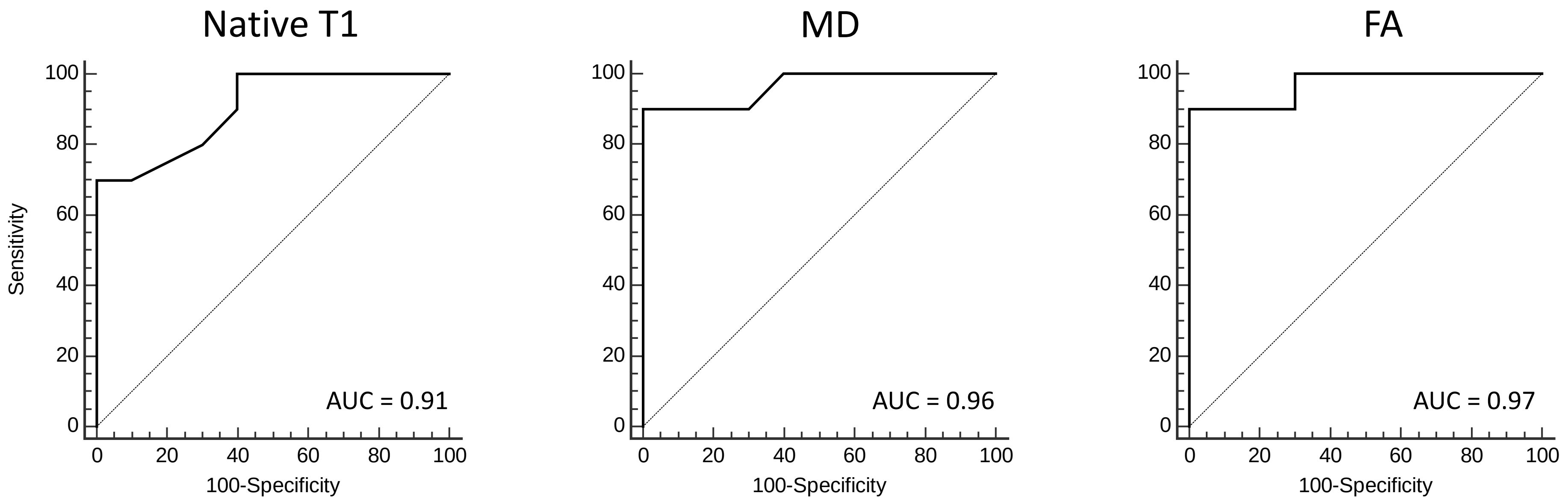

	Native T1	MD	FA
Diagnostic criterion	> 1085 ms	> 1.51 $10^{-3}\frac{s}{mm^2}$	< 0.29
95% CI AUC	0.69 – 0.99	0.77 – 1.00	0.78 – 1.00
Sensitivity	70 %	90 %	90 %
Specificity	100 %	100 %	100 %

Abbreviations: AUC, area under the curve; CI, confidence interval; FA, fractional anisotropy; MD, mean diffusivity
